# Borate‐Ion‐Stimulated Macrophages Promote Osteogenic Differentiation of Mesenchymal Stem Cells

**DOI:** 10.1002/adhm.202502570

**Published:** 2025-09-09

**Authors:** Kazumasa Ikedo, Hiroki Hatakeyama, Sayaka Oguri, Akiko Obata, Toshihiro Kasuga

**Affiliations:** ^1^ Graduate School of Engineering Nagoya Institute of Technology Gokiso‐cho, Showa‐ku Nagoya 466–8555 Japan

**Keywords:** borate ion, macrophages, mesenchymal stem cells, mineralization

## Abstract

Immune cells, such as macrophages, stimulated by several types of inorganic ions released from bioactive glasses secrete cytokines that promote and inhibit bone formation. In this study, the effects of borate‐ion‐stimulated mouse macrophages (RAW264) on the osteogenic differentiation of mouse bone marrow–derived mesenchymal stem cells (KUSA‐A1) are investigated. KUSA‐A1 is cultured with a borate‐ion‐containing medium and RAW264‐conditioned medium, which contained the secretome released from boron‐stimulated RAW264, and its osteogenic differentiation is evaluated. Results indicated that the secretome of RAW264 stimulated by more than 3 mg L^−1^ borate ions promoted the expression level of Gla‐osteocalcin in cells, and that of RAW264 stimulated by more than 10 mg L^−1^ borate ions promoted collagen production. Borate ions inhibited KUSA‐A1 calcification, whereas the RAW264‐conditioned medium promoted it. On the contrary, borate ions promoted gene expression levels in RAW264 cells, including anti‐inflammatory cytokine‐related and bone morphogenetic protein‐2‐related genes. Therefore, the immune response of RAW264 stimulated by borate ions can promote the osteogenic differentiation and mineralization of KUSA‐A1, indicating that the ability to elute borate ions will be useful in the design of glass materials for bone regeneration.

## Graphical abstract

The immune responses of mouse macrophages stimulated by borate ions promote the osteogenic differentiation and mineralization of mouse bone marrow–derived mesenchymal stem cells in an ion‐dose‐dependent manner by providing cytokines, including BMP‐2. Therefore, the ability to elute borate ions is useful in the design of glass materials for bone regeneration.

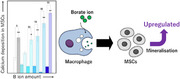

## 1 Introduction

The skeletal system is closely related to the immune system, and the responses of immune cells play an important role in bone remodeling and bone diseases.^[^ ^1^, ^2^, ^3^, ^4^ ^]^ Immune cells, such as macrophages, initially respond to external foreign substances in the body. They recognize leachates and other substances from materials implanted in the body and initiate immune responses.^[^ ^5^ ^]^ In this process, macrophages polarize into two different phenotypes, M1 or M2.^[^ ^6^ ^]^ M1 and M2 macrophages provide inflammatory and anti‐inflammatory cytokines, respectively.^[^ ^7^, ^8^ ^]^ Inflammatory cytokines play an important role in the elimination of bacteria and viruses. In addition, they promote bone resorption and inhibit bone formation. For example, inflammatory cytokines inhibit the differentiation and mineralization of human periosteum‐derived osteoblasts, as well as the proliferation, differentiation, and collagen synthesis of mouse mesenchymal stem cells (mMSC).^[^ ^8^, ^9^, ^10^, ^11^, ^12^ ^]^ However, some reports have revealed that inflammatory cytokines promote bone formation, indicating that their effects vary depending on their dose, cell type, timing of administration, etc.^[^ ^13^, ^14^ ^]^ On the contrary, M2 macrophages express anti‐inflammatory cytokines.^[^ ^7^, ^8^ ^]^ Anti‐inflammatory cytokines secreted by M2 macrophages promote the mineralization of mouse osteoblast‐like cells (MC3T3‐E1 cells), as well as the differentiation and mineralization of mMSC.^[^ ^15^, ^16^ ^]^ In addition, bone morphogenetic protein‐2 (BMP‐2) released by M2 macrophages promotes bone formation.^[^ ^17^ ^]^ Thus, immune cells have attracted attention because of their important roles in bone formation, and some biomaterials with immunomodulatory functions have been developed in recent years as a novel type of material for tissue regeneration.^[^ ^18^ ^]^

The effects of various inorganic ions, especially ions released from bioactive glasses and ceramics, on bone formation through immune cell responses have been previously studied.^[^ ^19^ ^]^ In this study, we focused on borate ions, which have been reported to influence bone formation and suppress the expression of inflammatory cytokines in immune responses. Boron is a trace element that can be found in the body, and boron intake has been reported to be associated with bone mineralization in vitamin D‐deficient chicks, indicating its importance to animals.^[^ ^20^ ^]^ Furthermore, in 1987, boron intake was found to suppress calcium excretion in postmenopausal women, leading to the recognition of boric acid ions as inorganic ions that promote bone formation.^[^ ^21^ ^]^ Borate ions promote the calcification of MC3T3‐E1 cells and rat bone marrow–derived mesenchymal stem cells (rBMSC) at 1–10 ng mL^−1^ and the differentiation of human bone marrow stromal cells at 10 and 100 ng mL^−1^ in vitro.^[^ ^22^, ^23^, ^24^ ^]^ Recently, borate ions were identified as inorganic ions that stimulate immune cells and suppress inflammatory responses.^[^ ^25^ ^]^ They can also inhibit the secretion of tumor necrosis factor‐α (TNF‐α), which is an inflammatory cytokine secreted by human monocyte–derived cells stimulated with lipopolysaccharides (LPS).^[^ ^26^ ^]^ The effects of boron‐containing biomaterials were also investigated. Calcium fructoborate inhibited the inflammatory cytokine secretion in LPS‐stimulated mouse‐derived macrophages (RAW264.7).^[^ ^27^ ^]^ The polarization of RAW264.7 into M2 macrophages and the secretion of anti‐inflammatory cytokines were enhanced on Ca_11_Si_4_B_2_O_22_ ceramic coatings compared with CaSiO_3_ coatings. Mesoporous bioactive glass nanoparticles incorporated with boron inhibited the secretion of anti‐inflammatory cytokines in mouse‐derived macrophage cells (J774a.1).^[^ ^28^, ^29^ ^]^ In addition, the effect of borate ions on the interaction between immune cells and bone‐forming cells has been previously reported. Boron‐loaded calcium silicate stimulated macrophages and MSCs, which contributed to the increased expression level of BMP‐2 and the activation of the BMP‐2‐related signaling pathway in MSCs.^[^ ^28^ ^]^ Despite these findings, few studies have specifically investigated the borate ion–mediated interactions between immune cells and osteogenic cells. Therefore, conducting further investigation is necessary to elucidate the role of borate ions in immune‐regulated bone formation and to develop novel materials with borate‐releasing abilities for use in bone tissue regeneration.

Previous studies have investigated only low concentrations of borate ions because borate ions above 1 mg L^−1^ have been reported to inhibit the growth of MC3T3‐E1 cells after 24 h of culture.^[^ ^22^ ^]^ In view of its actual application as a material for bone repair, handling such small amounts is difficult. From this perspective, the effects of high borate ion concentrations should also be investigated. Therefore, this study aimed to clarify the effects of various concentrations of borate ions, including more than 1 mg L^−1^, on the osteogenic process of mMSC through immune responses by mouse‐derived macrophage cells (RAW264).

## 2 Results

### 2.1 Proliferation of RAW264

**Figure 1:**
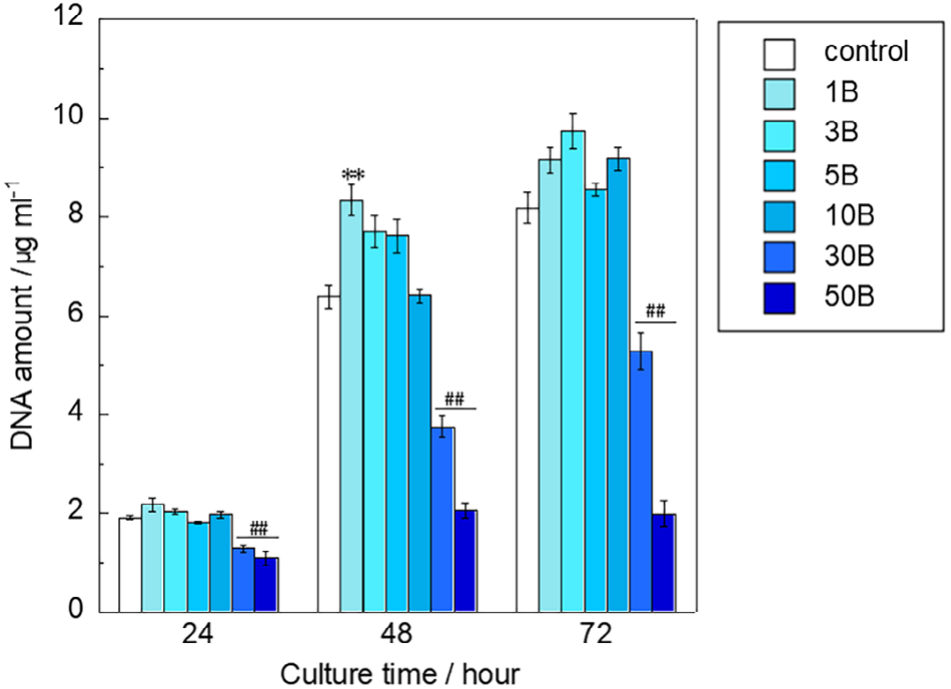
Amount of DNA in RAW264 cultured with B‐containing medium–high (*n* = 4, ^**^, ^##^: *p *< 0.01 vs control).

**Figures** **1** and S1 (Supporting Information) show the amount of deoxyribonucleic acid (DNA) and the metabolic activity, respectively, in RAW264 after culturing in borate ion‐containing medium–high (B‐containing medium–high). Because both results show a similar trend, we believe that the DNA quantification results accurately reflect cell proliferation. The amount of DNA for 30B and 50B (B‐containing medium–high with 30 and 50 mg L^−1^ of borate ions, respectively) was significantly lower than that for the control at all time points, whereas that for 1B was significantly higher at 48 h. As the values of 50B showed almost no change during culture, the B‐containing medium–high/low with 50 mg L^−1^ of borate ions was not used for subsequent experiments. The values for the 30B samples are significantly lower than those of the control samples; however, they do show a slight increase over the 72‐h culture period. In addition, the cellular response observed in the 30B samples is markedly different from that of the 1B‐10B samples. Since the aim of this study is to investigate the effects of secretions from the cells, we considered that even if the cell number decreased, the 30B samples might be expressing different secretions compared to the 1B‐10B samples. Therefore, we used the 30B samples in other culture experiments.

### 2.2 Osteocalcin Expression

**Figure 2:**
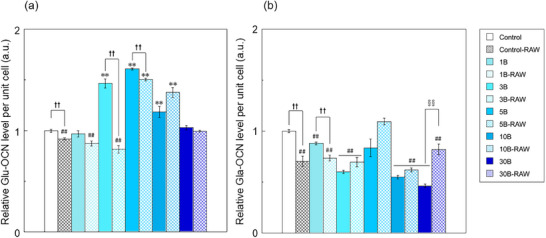
a) Glu‐OCN, b) Gla‐OCN expression per unit cell of KUSA‐A1 cultured with the B‐containing mixture and RAW‐conditioned media (value of control as 1, *n* = 3, ^**^, ^##^: *p *< 0.01 versus control,††: *p* < 0.01).

**Figure** **2** shows the expression level of osteocalcin (OCN) per unit cell of mouse bone marrow–derived mesenchymal stem cells (KUSA‐A1) when cultured with a mixture of B‐containing medium–high and –low (B‐containing mixture) and a medium containing the secretome of RAW264 after stimulation with borate ions (RAW‐conditioned medium). In this experiment, the expression level of γ‐carboxylated osteocalcin (Gla‐OCN) and uncarboxylated osteocalcin (Glu‐OCN) was measured. The code for the samples cultured with the RAW conditioned medium was “‐RAW” at the bottom of each sample code. For Glu‐OCN, 3B, 5B, 5B‐RAW, 10B, and 10B‐RAW showed significantly higher expression levels than the control, whereas control‐RAW, 1B‐RAW, and 3B‐RAW showed significantly lower expression levels than the control. The control‐RAW values were significantly lower than those of the control for Glu‐OCN and Gla‐OCN. 3B‐RAW, 5B‐RAW, and 30B‐RAW showed lower Glu‐OCN expression levels than 3B, 5B, and 30B, respectively, whereas 3B‐RAW, 5B‐RAW, and 30B‐RAW showed higher Gla‐OCN expression levels than 3B, 5B, and 30B, respectively.

### 2.3 Collagen Production by KUSA‐A1 Cells

**Figure 3:**
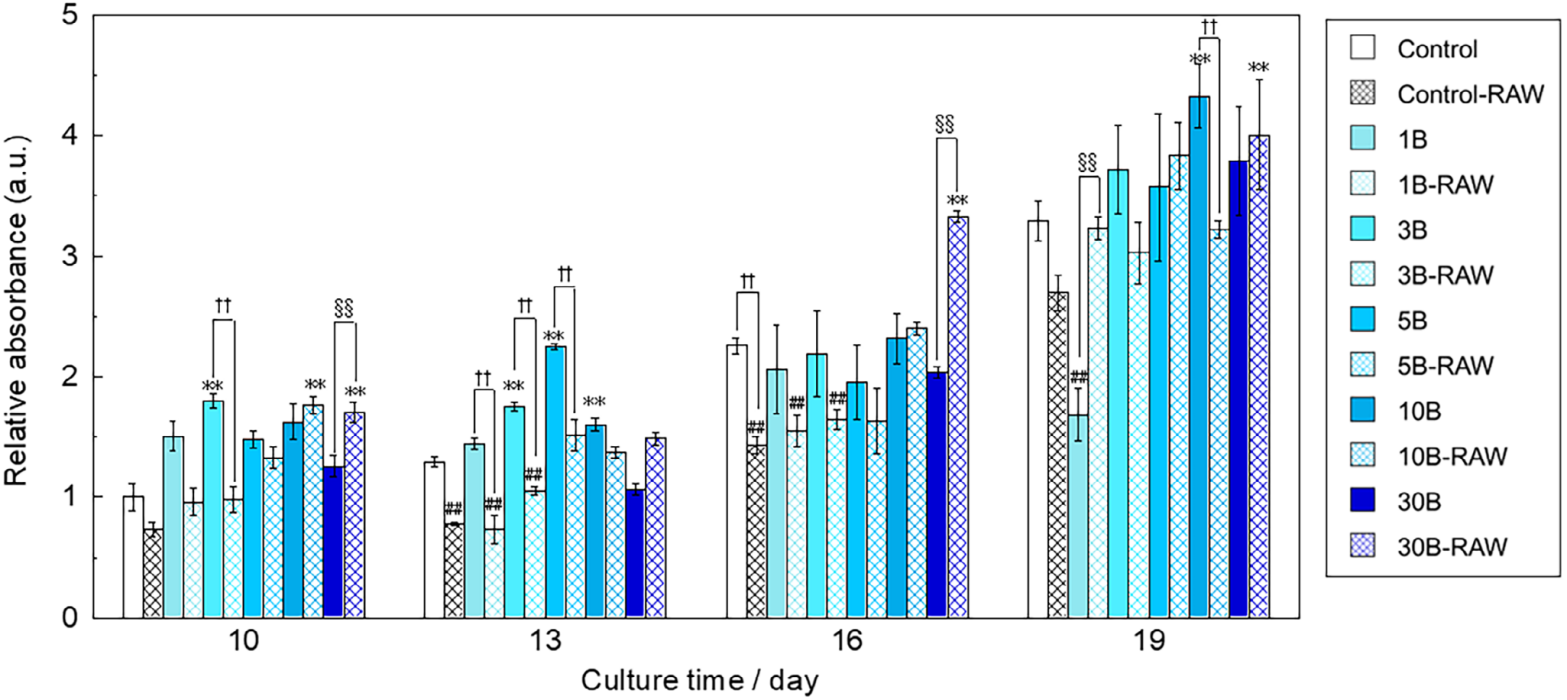
Collagen production of KUSA‐A1 cultured with the B‐containing mixture and RAW‐conditioned media (value of control at 10 days as 1, *n* = 4, ^**^, ^##^: *p *< 0.01 versus control,§§, ††: *p* < 0.01).

**Figure** **3** shows the collagen production per unit cell of KUSA‐A1 when cultured in a B‐containing mixture and a RAW‐conditioned medium. After 10 days of culture, the collagen production of 3B, 10B‐RAW, and 30B‐RAW was significantly higher than that of the control. In addition, the collagen production of 3B‐RAW was significantly lower than that of 3B, and the collagen production of 30‐RAW was significantly higher than that of 30B. The collagen production of B‐containing mixture was higher than control. After 13 days of incubation, control‐RAW, 1B‐RAW, and 3B‐RAW showed significantly lower collagen production than the control, whereas 3B, 5B, and 10B showed significantly higher collagen production than the control. 1B‐RAW, 3B‐RAW, and 5B‐RAW showed significantly lower collagen production than 1B, 3B, and 5B respectively. After 16 days of incubation, control‐RAW, 1B‐RAW, and 3B‐RAW showed significantly lower collagen production than the control. Control‐RAW showed significantly lower collagen production than the control, whereas 30B‐RAW showed significantly higher collagen production than 30B. Moreover, the collagen production of 30B‐RAW was significantly higher than that of the control. After 19 days of incubation, all samples exhibited higher values compared with those observed at 16 days, except for 1B, which showed significantly lower values than the control and 1B‐RAW. 10B and 30B‐RAW showed significantly higher collagen production than the control. 10B‐RAW showed a significantly lower collagen production than 10B. Thus, the B‐containing mixture with a smaller amount of borate ions enhanced collagen synthesis in the early stage, and the RAW‐conditioned medium with a large amount of ions promoted collagen synthesis compared with the B‐containing mixture.

### 2.4 Calcium Deposition

**Figure 4:**
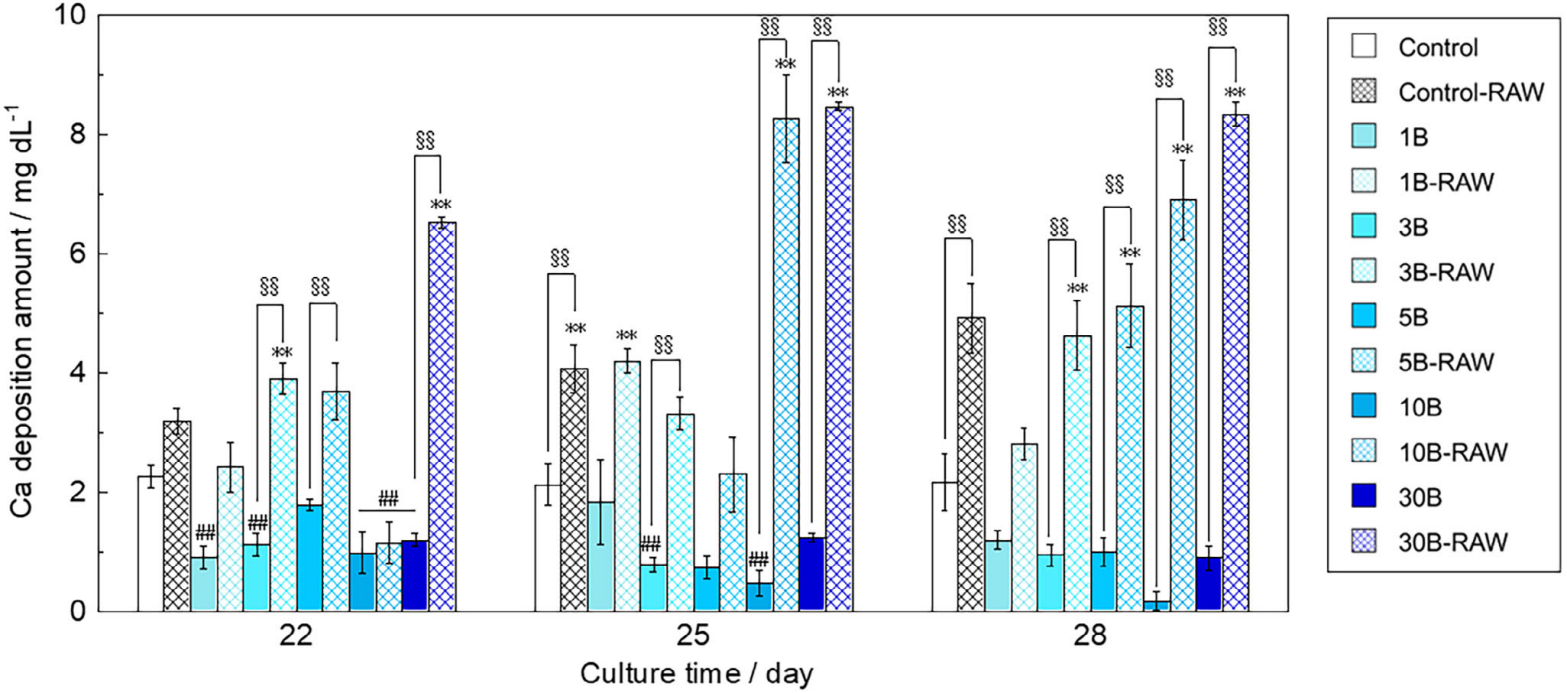
Calcium deposition of KUSA‐A1 cultured with the B‐containing mixture and RAW‐conditioned media (*n* = 4, ^**^, ^##^: *p *< 0.01 V.S. control,§§, ††: *p* < 0.01).

**Figure** **4** shows the calcium deposition of KUSA‐A1 cultured in a B‐containing mixture and RAW‐conditioned media. After 22 days of culture, the calcium deposition level of 1B, 3B, 10B, 10B‐RAW, and 30B was significantly lower than that of the control, whereas 3B‐RAW and 30B‐RAW showed significantly higher calcium deposition level than the control. Furthermore, 3B‐RAW, 5B‐RAW, and 30B‐RAW exhibited significantly higher calcium deposition level than 3B, 5B, and 30B, respectively. After 25 days of incubation, control‐RAW, 1B‐RAW, 10B‐RAW, and 30B‐RAW showed significantly higher calcium deposition level than the control. Furthermore, 3B‐RAW, 10B‐RAW, and 30B‐RAW exhibited significantly higher calcium deposition level than 3B, 10B, and 30B, respectively. After 28 days of incubation, all RAW‐conditioned media with borate ion supplementation, except for 1B‐RAW, showed significantly higher calcium deposition level than B‐containing mixtures. Thus, the RAW‐conditioned media enhanced KUSA‐A1 mineralization in a borate ion‐dose‐dependent manner.

### 2.5 Gene Expression in RAW264

**Figure 5:**
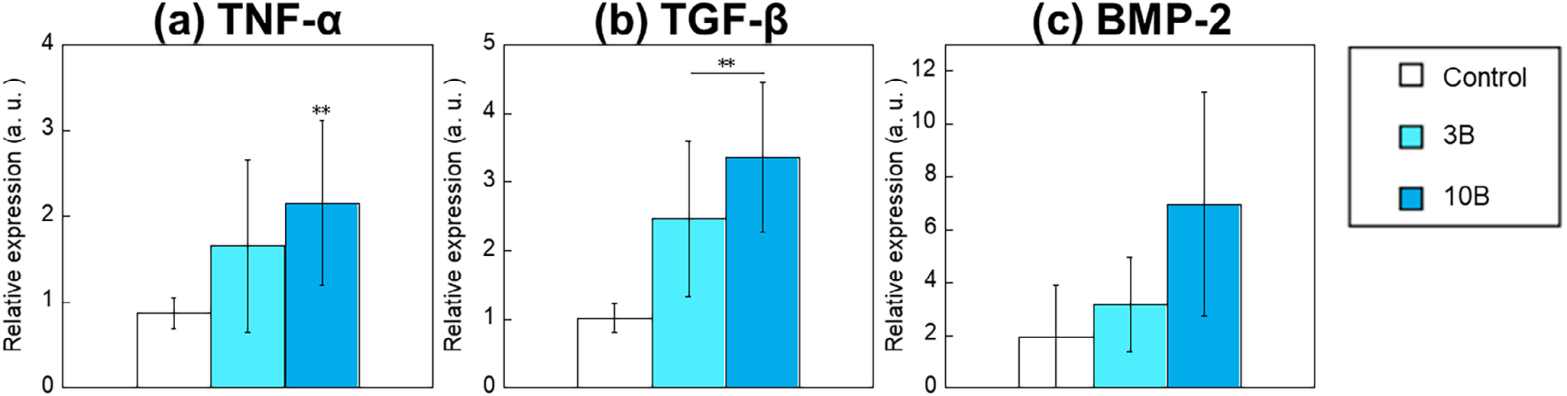
Gene expression of a) TNF‐α, b) TGF‐β, and c) BMP‐2 of RAW264 cultured with B‐containing medium–high for 72 h (*n* = 3, ^**^, ^##^: *p *< 0.01 V.S. control).

In measuring gene expression, 3 and 10 mg L^−1^ were used as the B ion concentration conditions because no cytotoxicity was observed for them, and 3B‐RAW and 10B‐RAW exhibited significantly higher calcium deposition levels compared with the control. **Figure** **5** shows the expression levels of the genes related to TNF‐α, transforming growth factor‐β (TGF‐β), and BMP‐2 of RAW264 after culturing in the B‐containing medium–high for 72 h. The expression levels of other genes are summarized in Figure S2 (Supporting Information). Because they include the result values obtained from samples with smaller than 3. All gene expression levels of 3B and 10B were higher than those of the control. The levels of TNF‐α, TGF‐β, and BMP‐2 were higher in the 10B group than in the 3B group, but no significant difference was found between the two groups. On the other hand, 10B possessed significantly higher levels of TNF‐α and TGF‐β than the control, and 3B did significantly higher levels of TGF‐β than the control.

### 2.6 Mitogen‐Activated Protein Kinase (MAPK) in RAW264

**Figure 6:**
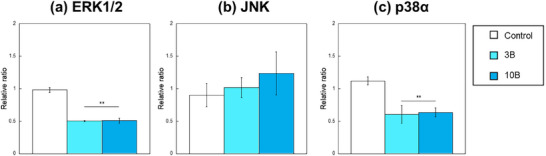
Phosphorylation rate of a) ERK1/2, b) JNK, and c) p38 in RAW264 cultured with B‐containing medium–high for 72 h (*n* = 4, ^**^, ^##^: *p *< 0.01 V.S. control).

In examining the effects of borate ions on the intracellular reaction pathway of RAW264, MAPK activity was evaluated after 72 h of culture with the B‐containing medium–high. **Figure** **6** shows the MAPK activation level, which was measured on the basis of the phosphorylation rate of three parts of the pathway: extracellular signal‐regulated kinase 1/2 (ERK1/2), c‐Jun N‐terminal kinase (JNK), and p38α. Borate ions significantly suppressed the phosphorylation rate of ERK1/2 and p38α. Although no significant difference was found, the rate of JNK increased with boron concentrations.

## 3 Discussion

Borate ions influenced the proliferation and expression of some genes in RAW264 cells in a dose‐dependent manner. The results of the DNA amount measurement indicated that higher concentrations of borate ions, such as 30 and 50 mg L^−1^, significantly decreased the proliferation ability of RAW264. By contrast, 1 mg L^−1^ significantly increased the proliferation ability of RAW264 after culturing for 48 h. Furthermore, the amount of DNA for 1, 3, and 5 mg L^−1^ was higher than that for the control at both 48 and 72 h (Figure 1). Based on previous reports, boron influences the proliferation, apoptosis, and immune function of lymphocytes via the ERK1/2 signaling pathway.^[^ ^30^ ^]^ In particular, 0.3 mmol L^−1^ (3.2 mg L^−1^) of borate ions promotes the proliferation of human cervical carcinoma cells by activating the ERK1/2 pathway, whereas 5 mmol L^−1^ (54 mg L^−1^) of borate ions suppresses such proliferation.^[^ ^31^ ^]^ Similarly, in the present study, the activation of the ERK1/2 pathway was significantly deteriorated by 3 and 10 mg L^−1^ of borate ions. Therefore, the reduction in the proliferation of RAW264 for 30B and 50B might be induced by inhibiting the activation of the ERK1/2 pathway because of the large amount of borate ions. However, the phosphorylation rate of ERK1/2 for 3B was lower than that of the control (Figure 6a), whereas the proliferation was enhanced. This result may indicate that other pathways influence the proliferation of RAW264 cultured with smaller amounts of borate ions, such as 3B.

Borate ions have been reported to promote the gene expression of OCN and COL and to induce matrix mineralization.^[^ ^22^, ^23^, ^24^ ^]^ OCN is an important protein in bone metabolism and is exclusively produced by osteoblasts in the form of Glu‐OCN. Glu‐OCN is carboxylated by γ‐glutamylcarboxylase (GGCX) and converted to Gla‐OCN.^[^ ^32^ ^]^ Gla‐OCN traps calcium ions and plays a role in inducing mineralization.^[^ ^33^ ^]^ Gla‐OCN is also decarboxylated during bone resorption and released into the blood as Glu‐OCN.^[^ ^34^ ^]^ Glu‐OCN is transported to pancreatic beta cells, and it stimulates insulin secretion, which promotes bone formation by osteoblasts.^[^ ^35^ ^]^ Bone remodeling is a tightly regulated process in which bone resorption and formation are intricately coupled. Glu‐OCN secreted during the bone resorptive phase contributes to a feedback loop that stimulates new bone formation by osteoblasts. Therefore, regulating OCN secretion is crucial for maintaining proper bone remodeling. The results of the Glu‐OCN measurements (Figure 2) indicated that the values for 3B, 5B, 5B‐RAW, 10B, and 10B‐RAW were significantly higher than those of the control. Less than 1 mg/L of borate ions was reported to promote OCN production in MC3T3‐E1 cells and human bone marrow–derived mesenchymal stem cells (hBMSC).^[^ ^22^, ^23^, ^24^ ^]^ The present results confirm that borate ions above 1 mg L^−1^ also show similar results. Although the detailed process of OCN production in osteoblasts has not been elucidated, transcription factors such as runt‐related transcription factor 2, core binding factor α1, and osteoblast‐specific factor 2 are involved in the expression of mouse osteocalcin gene 1, osteocalcin gene 2, and osteocalcin‐related genes.^[^ ^36^, ^37^ ^]^ Borate ions may also stimulate these transcription factors. The results of the Glu‐OCN and Gla‐OCN measurements showed that control‐RAW was significantly lower than the control. Therefore, cytokines secreted by RAW264 inhibit osteoblast differentiation. 3B‐RAW, 5B‐RAW, and 30B‐RAW showed lower Glu‐OCN expression levels than 3B, 5B, and 30B, respectively, but showed higher Gla‐OCN expression levels. This result indicated that the RAW264 cells, which were stimulated by borate ions, secreted cytokines that promoted the carboxylation of Glu‐OCN. Carboxylation by GGCX is part of the vitamin K cycle through the vitamin K epoxide reductase complex subunit 1 and is known to be dependent on vitamin K.^[^ ^38^ ^]^ In addition, Gla‐OCN played a role in assisting mineralization, which indicated that cytokines secreted from RAW264 stimulated by borate ions can promote the early stages of KUSA‐A1 mineralization.

Collagen is synthesized by osteoblasts before mineralization.^[^ ^39^ ^]^ Collagen type I, which is particularly abundant in bone, has a triple helical structure of polypeptides that come together to form fibers.^[^ ^40^ ^]^ These fibers react with collagen type III and type IV, as well as other proteins, to create a strong fiber structure, which improves bone strength. Apart from these mechanical properties, collagen type I also contributes to bone mineralization.^[^ ^41^ ^]^ After 10 days of culture, 3B, 10B‐RAW, and 30B‐RAW showed significantly higher collagen type I production than the control. In addition, 3B showed significantly higher collagen production than the control after 13 days. Therefore, borate ion concentration below 3 mg L^−1^ promoted collagen type I production. This result is similar to that of a previous study; 1 mg L^−1^ of borate ions promotes collagen production in MC3T3‐E1 cells.^[^ ^9^ ^]^ After 10, 13, and 16 days of culture, control‐RAW, 1B‐RAW, 3B‐RAW, and 5B‐RAW produced lower amounts of collagen than the control, 1B, 3B, and 5B, respectively. By contrast, 30B‐RAW produced higher amounts of collagen than 30B. This result indicated that cytokines secreted by RAW264 stimulated by 5 mg L^−1^ of borate ions or less inhibit the production of collagen type I, whereas those secreted by RAW264 stimulated by 30 mg L^−1^ of borate ions promote such production. Many research groups have conducted studies on the effects of cytokines on collagen production. For example, TGF‐β was reported to promote the production of collagen type I, but inflammatory cytokines inhibit such production.^[^ ^42^, ^43^, ^44^ ^]^ In the present study, considering that the expression level of TGF‐β was higher for 10B than for 3B, 30B‐RAW may promote the production of collagen type I by enhancing the expression level of TGF‐β in RAW264.

As shown in Figure 4, after 22 days of culture, all samples cultured in the B‐containing mixture showed lower calcium deposition than the control, indicating that borate ions inhibited the mineralization of KUSA‐A1. In previous reports, borate ion concentrations of 10 µg L^−1^ or less promoted hBMSC mineralization, whereas borate ion concentrations above 100 µg L^−1^ inhibited hBMSC mineralization,^[^ ^11^ ^]^ which is consistent with the present results. On the contrary, borate ions below 1 mg L^−1^ promoted the mineralization of MC3T3‐E1 cells, but nothing above 1 mg L ^−1^has been reported.^[^ ^9^ ^]^ In the case of rBMSC mineralization, borate ions at 6 mg L^−1^ showed similar results to the control.^[^ ^10^ ^]^ Thus, the results are different from those of the present study; however, they are difficult to compare because an osteogenesis‐inducing medium was used in the previous report. After 28 days of culture, all samples cultured with RAW‐conditioned media, except for control‐RAW and 1B‐RAW, showed significantly higher calcium deposition than the control. In addition, all RAW‐conditioned media showed higher values than the B‐conditioned mixture at the same B concentration, indicating that mineralization was enhanced by the secretome released by RAW264 stimulated by borate ions. Furthermore, the difference in calcium deposition between *x*B‐RAWs and *x*B was more pronounced at higher borate ion concentrations. This result indicated that the enhancements in mineralization by the secretome released by RAW264 exhibit a dose‐dependent effect on borate ions.

The gene expression in RAW264 cells after being stimulated with borate ions was evaluated to clarify whether the cytokines released from them were related to the enhancement of the osteogenic differentiation and mineralization of KUSA‐A1. 10B exhibited higher gene expression levels than 3B for each individual gene (Figure 5). In particular, the expression levels of BMP‐2 and TGF‐β, which have been reported to promote bone formation, were upregulated more by 10B than by 3B. BMP‐2 and TGF‐β1 secreted by M2 macrophages have been reported to stimulate Smad‐related signaling and promote bone formation.^[^ ^45^, ^46^ ^]^ In the present study, the production of Glu‐OCN and collagen (on days 10, 13, 16, and 19) and calcium deposition (on days 25 and 28) were higher in 10B‐RAW than in 3B‐RAW. Thus, borate ions can enhance the production of cytokines that induce bone formation in RAW264 in a dose‐dependent manner, which promotes KUSA‐A1 mineralization. Interestingly, the increase in the gene expression level of BMP‐2 was higher than that of the other genes after culturing with borate ions. To the best of our knowledge, no studies have reported on the promotion of BMP‐2 production in macrophages by inorganic ions, except for one report on calcium ions. The mRNA expression level and secretion of BMP‐2 in J774A.1 cells were significantly increased when incubated in media with calcium ion concentrations up to 14 mmol L^−1^ (200 mg L^−1^).^[^ ^47^ ^]^

Although M1 macrophages have been reported to inhibit osteogenesis, some reports have shown that they can promote osteogenesis.^[^ ^48^ ^]^ Oncostatin M belonging to the IL‐6 family has also been reported to promote bone formation.^[^ ^49^ ^]^ A low concentration of TNF‐α, which is an inflammatory cytokine, cannot inhibit bone formation.^[^ ^44^, ^50^ ^]^ Therefore, although borate ions enhance the production of some inflammatory cytokines in macrophages, they mainly induce their polarization toward M2 type, which enhances bone formation in mesenchymal stem cells.

MAPKs have been reported to be involved in the production of cytokines;^[^ ^51^ ^]^ thus, borate ions may influence the gene expression of RAW264 by stimulating their MAPKs. In the present study, considering the differences in the activation rate among ERK1/2, JNK, and p38α (Figure 6), the MAPK activity might be involved in the observed changes in the gene expression of cytokines. Given that JNK is involved in BMP‐2‐induced osteoblastic differentiation, the enhanced calcium deposition observed for most of the RAW264 cultured with B ions might be due to the activation of JNK induced with the ions (Figure 6b).^[^ ^52^ ^]^ On the contrary, the ions suppressed the phosphorylation of ERK1/2 and p38α.

A study on the ostrich thymus reported that increased boron dosage leads to a decrease in phosphorylated ERK expression, thereby increasing JNK expression to prevent excessive T‐cell activation.^[^ ^53^ ^]^ These results are similar to those observed in this study. Furthermore, the ERK inhibitor PD98059 has been reported to increase TNF‐α production when pretreated at low concentrations (0.37–3.33 µm) in human peripheral blood monocytes for 30 min.^[^ ^54^ ^]^ In addition, the expression level of TNF‐α in macrophages correlates with p38 expression.^[^ ^55^, ^56^, ^57^ ^]^ Therefore, the inhibition of ERK and p38α phosphorylation found in our experiment may be related to the increased expression level of TNF‐α‐related genes. Although ERK and p38α have been reported to implicate TGF‐β and BMP‐2 production, our experimental findings diverged, indicating that other pathways may be at play.^[^ ^58^, ^59^ ^]^ Aspects regarding the intracellular pathways, involving borate ions, remain unknown; thus, further investigation is needed in the future.

Although various studies have reported the effects of borate ions on bone formation, the amount of ions in these reports was very small, which caused difficulty in the application of ion effects to material design, such as bioactive borate glasses. In addition, the mechanism by which the secretome of macrophages stimulated by borate ions influences the bone‐forming ability of mesenchymal stem cells remains unclear. We believe that the present study is the first to report that large amounts of borate ions (>1 mg L^−1^) significantly enhance the mineralization of mesenchymal stem cells by stimulating macrophages, which is due to the enhanced production of cytokines related to bone formation in macrophages. Furthermore, these phenomena exhibited a borate ion‐dose‐dependent manner. Although silicate and phosphate glasses have received considerable attention as bone substitutes, this study has newly discovered that borate glasses are very useful. These findings of this study will contribute to the design of novel bioactive glasses for bone regeneration.

## 4 Conclusion

This study aimed to determine the effects of various concentrations of borate ions on the osteogenic process of mesenchymal stem cells through the immune response of macrophages. Using a macrophage‐conditioned medium, the effect of the immunoreactivity of RAW264 after stimulation with borate ions on osteogenic differentiation was evaluated, inducing the production of collagen and calcium deposition in KUSA‐A1 cells. Interestingly, the medium containing borate ions inhibited calcium deposition in KUSA‐A1 cells regardless of the borate ion concentration, whereas the RAW‐conditioned medium promoted calcium deposition. In particular, the calcium deposition of the secretome of RAW264 cells stimulated with 10 mg L^−1^ of borate ions or higher was markedly enhanced. The expression level of genes related to BMP‐2 in RAW264 was particularly enhanced by borate ions in an ion‐dose‐dependent manner, indicating that the immune response of RAW264 stimulated by appropriate amounts of borate ions greatly enhances the mineralization of KUSA‐A1 cells. These results indicate that materials such as borate glasses, which provide appropriate amounts of borate ions, are useful for bone regeneration via the stimulation of immune cells.

## 5 Experimental Section

### Preparation of the Culture Media

Dulbecco's modified Eagle medium–high glucose GlutaMAX Supplement (DMEM high glucose, Life Technologies) and Dulbecco's modified Eagle medium–low glucose GlutaMAX Supplement (DMEM low glucose, Life Technologies) were used as culture media for RAW264 and mouse bone marrow–derived mesenchymal stem cells (KUSA‐A1), respectively, after being supplemented with 10 vol.% foetal bovine serum (Biowest) and 1 vol.% of penicillin–streptomycin (Life Technologies). These media are hereafter indicated by “normal‐medium–high” and “normal–medium–low”, respectively.

In preparing the borate ion‐containing media, boric acid (H_3_BO_3_, Fujifilm Wako Pure Chemicals) was added to the normal‐medium–high and the normal–medium–low and sterilized by filtration through a 0.2‐µm CA filter (Minisart Syringe Filter, Sartorius), which were denoted by “B‐containing medium–high” and “B‐containing medium–low”, respectively. The borate ion concentrations were set to 1, 3, 5, 10, 30, and 50 mg L^−1^. The concentration of borate ions in the prepared B‐containing medium (high/low) was determined using inductively coupled plasma atomic emission spectrometry (ICPS‐7510, Shimadzu Corporation). The code of the samples cultured with the B‐containing medium–high/low was explained using its B ion concentration, such as “1B”. The culture medium without ion supplementation was used as the “control”.

### Preparation of the RAW‐ Conditioned Media

RAW264 suspensions (2.0 × 10^4^ cells mL^−1^) were prepared using the normal‐medium–high. The suspension (500 µL) was seeded in 24‐well plates (Thermo Scientific) and cultured in a CO_2_ incubator (CO_2_ concentration 5%, 37 °C, MCO‐17AIC, Sanyo Denki). After 24 h of culture, the culture medium in each well was exchanged with the B‐containing medium–high and incubated at 37 °C. After 72 h of incubation, the culture medium was collected, and the supernatant was extracted after centrifugation. The resulting supernatant was mixed with B‐containing medium–low with the same B concentration at a 2:1 ratio (in volume). The ratio was selected on the basis of the culture test conducted in the previous study.^[^ ^19^ ^]^ The resulting medium is hereafter referred to as the “RAW‐conditioned medium” and the code for the samples cultured with the RAW‐conditioned medium was “‐RAW” at the bottom of each sample code, such as 1B‐RAW. The mixture of the normal‐medium–high and the normal–medium–low in a 2:1 ratio with and without ion supplementation was indicated as the “B‐containing mixture” and “normal mixture”, respectively (they contained no secretome of RAW264).

### Evaluation of the Proliferation Ability of RAW264

First, the effects of the B ion concentration on the proliferation of RAW264 were evaluated by measuring their DNA amounts during the 72‐h culture. Next, 100 µL of the RAW264 suspension, which was prepared using the normal‐medium–high set to 2.0 × 10^4^ cells mL^−1^, was placed in 96‐well plates (Thermo Scientific) and cultured in a CO_2_ incubator. After 24 h, the culture medium was removed from the wells, and B‐containing medium–high was added to the wells. Subsequently, the medium was incubated for 24, 48, and 72 h in a CO_2_ incubator.

The amount of DNA was measured using a DNA Quantitation Kit, Fluorescence Assay (Sigma‐Aldrich) in accordance with the manufacturer's instructions (*n* = 4). In brief, the medium in each well was removed, and the wells were rinsed two times with phosphate‐buffered saline (PBS, pH 7.4). Then, 50 µL of ultrapure water was added to the wells, and the cells were lysed by freeze‐thawing the well plate six times. Subsequently, 40 µL of the resulting solution was transferred to another 96‐well plate and mixed with 40 µL of the bisbenzimide H 33 258 solution (1 µg mL^−1^). The amount of DNA in each well was estimated by measuring the fluorescence intensity at excitation and emission wavelengths of 360  and 460 nm, respectively, using a multimode plate reader (EnSpire, PerkinElmer).

### Culture Tests Using KUSA‐A1—Cell Culture

The suspensions of KUSA‐A1 prepared using the normal–medium–low method were seeded in 96‐well plates (100 µL, 2.0 × 10^3^ cells mL^−1^) and cultured in a CO_2_ incubator. After 24 h, the medium in each well was replaced with the B‐containing mixture or RAW‐conditioned medium and then incubated for 10, 13, 16, 19, 22, 25, and 28 days in a CO_2_ incubator. The medium was replaced every 3 days. The normal mixture was used as the culture medium for the “control”.

### Culture Tests Using KUSA‐A1—Measurement of OCN Expression

The expression levels of Gla‐osteocalcin (Gla‐OCN) and Glu‐osteocalcin (Glu‐OCN) in the samples were measured using a Mouse Gla‐Osteocalcin High Sensitive EIA Kit (MK127, Takara Bio) and a Mouse Glu‐Osteocalcin High Sensitive EIA Kit (MK129, Takara Bio) after 19 days of culture, respectively, in accordance with the manufacturer's instructions (*n* = 3). The absorbance of the resulting samples was measured at a wavelength of 450 nm using a multimode plate reader. After the measurement, the samples were rinsed with PBS, and the amount of DNA was determined using the aforementioned method. The expression levels of Gla‐OCN and Glu‐OCN were divided by the amount of DNA to calculate the Gla‐OCN and Glu‐OCN expression per unit cell, respectively.

### Culture Tests Using KUSA‐A1—Measurement of Collagen Production

Collagen synthesis by KUSA‐A1 was measured using the Picrosirius Red Stain Kit (Polysciences) after 10, 13, 16, and 19 days of culture in accordance with the manufacturer's instructions (*n* = 4). The absorbance of the resulting samples was measured at a wavelength of 562 nm using a multimode plate reader.

### Culture Tests Using KUSA‐A1—Measurement of Calcium Deposition

Calcium deposition in the samples was measured after 22, 25, and 28 days of incubation using Calcium E‐Test Wako (Fujifilm Wako Pure Chemicals) in accordance with the manufacturer's instructions (*n* = 4). The absorbance at a wavelength of 610 nm was measured using a multimode plate reader.

### Measuring Macrophage Gene Expression Levels in RAW264

The expression of cytokine‐related genes associated with inflammation, anti‐inflammation, and bone formation in RAW264 was evaluated. RAW264 suspensions prepared using the normal‐medium–high method was seeded in 12‐well plates (1 mL, 2.0 × 10^4^ cells mL^−1^, *n* = 3) and cultured in a CO_2_ incubator. After 24 h, the culture medium was removed from each well, and B‐containing medium–high (3 and 10 mg L^−1^ of borate ions) was added to the well. After culturing for 72 h, the cells were treated with TRIzol reagent (Invitrogen), and total ribonucleic acid (RNA) was isolated from the cells in accordance with the manufacturer's instructions. Subsequently, complementary deoxyribonucleic acid (cDNA) was synthesized from the isolated RNA using the PrimeScript RT Master Mix (Takara Bio) for real‐time PCR analysis. Finally, cDNA, primers, and SYBR green mix (iTaq Universal SYBR Green Supermix, BIO‐RAD) were mixed in accordance with the manufacturer's instructions and examined using the intercalator method. The GAPDH (Takara Bio), TNF‐α (Takara Bio), NOS2 (Takara Bio), IL‐1β (Takara Bio), IL‐6 (Takara Bio), TGF‐β (Takara Bio), IL‐10 (Takara Bio), and BMP‐2 (Takara Bio) primers listed in **Table** **1** were used in this study.

**Table 1:** Primers used in this study.

Primer		Sequence
GAPDH	Forward	TGTGTCCGTCGTGGATCTGA
	Reverse	TTGCTGTTGAAGTCGCAGGAG
TNF‐α	Forward	ACTCCAGGCGGTGCCTATGT
	Reverse	GTGAGGGTCTGGGCCATAGAA
NOS2	Forward	CAAGCTGAACTTGAGCGAGGA
	Reverse	TTTACTCAGTGCCAGAAGCTGGA
IL‐1β	Forward	TCCAGGATGAGGACATGAGCAC
	Reverse	GAACGTCACACACCAGCAGGTTA
IL‐6	Forward	CCACTTCACAAGTCGGAGGCTTA
	Reverse	TGCAAGTGCATCATCGTTGTTC
TGF‐β	Forward	TTTCCGCTGCTACTGCAAGTC
	Reverse	GATAAGGCTTGGCAACCCAAGTAA
IL‐10	Forward	GCCAGAGCCACATGCTCCTA
	Reverse	GATAAGGCTTGGCAACCCAAGTAA
BMP‐2	Forward	TGACTGGATCGTGGCACCTC
	Reverse	CAGAGTCTGCACTATGGCATGGTTA

### Measuring MAPK Activation in RAW264

RAW264 (2.0 × 10^4^ cells mL^−1^, 1 mL, *n* = 4) were seeded using the normal medium and cultured in a CO_2_ incubator. After 24 h, the culture medium was removed from each well, and B‐containing medium–high (3 and 10 mg L^−1^ of borate ions) was added to the well. After culturing for an additional 72 h, the activation level of the MAPK‐related proteins (ERK1/2, JNK, and p38α) was evaluated by measuring the amount of total and phosphorylated MAPK‐related proteins using the Phospho‐ERK/JNK/P38α ELISA kit (Ray Biotech) in accordance with the manufacturer's instructions (*n* = 4). The absorbance at a wavelength of 450 nm was measured using a multimode plate reader. The results for each sample were obtained as relative values, with the value of the control sample set to 1. The ratio of phosphorylated protein to total protein was calculated to obtain the activation level of each protein.

### Statistical Analysis

One sample with the largest difference from the average value was excluded from each test to reduce the experimental error and obtain more accurate analytical results. Because some of the results in this study did not exhibit a normal distribution, a non‐parametric test was used to determine the statistical significance. Statistical differences in the data were determined using the Steel test (vs control) and the Steel‐Dwass test (for each sample). All significance tests were performed using R.

## Conflict of Interest

The authors declare no conflicts of interest.

## Supporting information



Supporting Information

## Data Availability

The data that support the findings of this study are available on request from the corresponding author. The data are not publicly available due to privacy or ethical restrictions.

## References

[1] J. Arron , Y. Choi , Nature 2000, 408, 535. 10.1038/35046196 11117729

[2] T. Hiroshi , Nat. Rev. Immunol. 2007, 7, 292.17380158

[3] L. J. Raggatt , N. C. Partridge , J. Bio. Chem. 2010, 285, 25103. 20501658 10.1074/jbc.R109.041087 PMC2919071

[4] J. Pajarinen , T. Lin , E. Gibon , Y. Kohno , M. Maruyama , K. Nathan , L. Lu , Z. Yao , S. B. Goodman , Biomaterials 2019, 196, 80. 29329642 10.1016/j.biomaterials.2017.12.025 PMC6028312

[5] Z. Xia , J. T. Triffitt , Biomed. Mater. 2006, 1, R1. 18458376 10.1088/1748-6041/1/1/R01

[6] M. Benoit , B. Desnues , J. L. Mege , J. Immunol. 2008, 181, 3733. 18768823 10.4049/jimmunol.181.6.3733

[7] D. Mosser , J. Edwards , Nat. Rev. Immunol. 2008, 8, 958. 19029990 10.1038/nri2448 PMC2724991

[8] S. Gordon , Nat. Rev. Immunol. 2003, 3, 23. 12511873 10.1038/nri978

[9] T. Kuroki , M. Shingu , Y. Koshihara , M. Nobunaga , Br. J. Rheumatol. 1994, 33, 224. 8156283 10.1093/rheumatology/33.3.224

[10] D. C. Lacey , P. J. Simmons , S. E. Graves , J. A. Hamilton , Osteoarthritis Cartilage 2009, 17, 735. 19136283 10.1016/j.joca.2008.11.011

[11] Y. Liu , L. Wang , T. Kikuiri , K. Akiyama , C. Chen , X. Xu , R. Yang , W. Chen , S. Wang , S. Shi , Nat. Med. 2011, 17, 1594. 22101767 10.1038/nm.2542 PMC3233650

[12] J. Lange , A. Sapozhnikova , C. Lu , D. Hu , X. Li , T. Miclau , R. S. Marcucio , J. Orthop. Res. 2010, 28, 778. 20041490 10.1002/jor.21061 PMC2858256

[13] H. Huang , N. Zhao , X. Xu , Y. Xu , S. Li , J. Zhang , P. Yang , Cell. Prolif. 2011, 44, 420. 21951285 10.1111/j.1365-2184.2011.00769.x PMC6495272

[14] S. Deshpande , A. W. James , J. Blough , A. Donneys , S. C. Wang , S. C. Cederna , S. R. Buchman , B. Levi , J. Surg. Res. 2013, 185, 278. 23972621 10.1016/j.jss.2013.06.063 PMC4489542

[15] F. Loi , L. A. Córdova , R. Zhang , J. Pajarinen , T. H. Lin , S. B. Goodman , Z. Yao , Stem. Cell. Res. Ther. 2016, 7, 15. 26801095 10.1186/s13287-016-0276-5 PMC4724110

[16] L. Gong , Y. Zhao , Y. Zhang , Z. Ruan , Ann. Clin. Lab. Sci. 2016, 46, 65. 26927345

[17] R. P. Pirraco , R. L. Reis , A. P. Marques , J. Tissue. Eng. Regen. Med. 2013, 7, 392. 22392849 10.1002/term.535

[18] K. Zheng , W. Niu , B. Lei , A. R. Boccaccini , Acta Biomater. 2021, 133, 168. 34418539 10.1016/j.actbio.2021.08.023

[19] O. Akiko , I. Makito , Y. Hikaru , K. Toshihiro , J. Biomed. Mater. Res. A 2025, 113, 37875.

[20] C. D. Hunt , F. H. Nielsen , Element Metabolism in Man and Animals, Springer‐Verlag, Heidelberg, Germany 1981, pp. 597.

[21] F. H. Nielsen , C. D. Hunt , L. M. Mullen , J. R. Hunt , FASEB J. 1987, 1, 394. 3678698

[22] S. S. Hakki , B. S. Bozkurt , E. E. Hakki , J. Trace. Elem. Med. Biol. 2010, 24, 243. 20685097 10.1016/j.jtemb.2010.03.003

[23] B. A. Movahedi Najafabadi , M. H. Abnosi , Cell J. 2016, 18, 62. 27054120 10.22074/cellj.2016.3988 PMC4819387

[24] X. Ying , S. Cheng , W. Wang , Z. Lin , Q. Chen , W. Zhang , D. Kou , Y. Shen , X. Cheng , F. A. Rompis , L. Peng , C. zhu Lu , Biol. Trace Elem. Res. 2011, 144, 306. 21625915 10.1007/s12011-011-9094-x

[25] F. H. Nielsen , Nutr. Rev. 2008, 66, 183. 18366532 10.1111/j.1753-4887.2008.00023.x

[26] J. Cao , L. Jiang , X. Zhang , X. Yao , C. Geng , X. Xue , L. Zhong , J. Trace Elem. Med. Biol. 2008, 22, 189. 18755394 10.1016/j.jtemb.2008.03.005

[27] R. I. Scorei , C. Ciofrangeanu , R. Ion , A. Cimpean , B. Galateanu , V. Mitran , D. Iordachescu , Biol. Trace Elem. Res. 2010, 135, 334. 19669712 10.1007/s12011-009-8488-5

[28] X. Lu , K. Li , Y. Xie , S. Qi , Q. Shen , J. Yu , L. Huang , X. Zheng , J. Biomed. Mater. Res. A 2019, 107, 12. 29781148 10.1002/jbm.a.36456

[29] K. Zheng , Y. Fan , E. Torre , P. Balasubramanian , N. Taccardi , C. Cassinelli , M. Morra , G. Iviglia , A. R. Boccaccini , Part. Part. Syst. Charact. 2020, 37, 2000054.

[30] S. Chen , H. Fan , Y. Pei , K. Zhang , F. Zhang , Q. Hu , E. Jin , S. Li , Biol. Trace Elem. Res. 2024, 202, 2688. 37737440 10.1007/s12011-023-03862-2

[31] M. Park , Q. Li , N. Shcheynikov , W. Zeng , S. Muallem , Mol. Cell 2004, 16, 331. 15525507 10.1016/j.molcel.2004.09.030

[32] P. V. Hauschka , J. B. Lian , D. E. Cole , C. M. Gundberg , Physiol. Rev. 1989, 69, 990. 2664828 10.1152/physrev.1989.69.3.990

[33] Q. Q. Hoang , F. Sicheri , A. J. Howard , D. S. C. Yang , Nature 2003, 425, 977. 14586470 10.1038/nature02079

[34] M. Ferron , J. Wei , T. Yoshizawa , A. Del Fattore , R. A. DePinho , A. Teti , P. Ducy , G. Karsenty , Cell 2010, 142, 296. 20655470 10.1016/j.cell.2010.06.003 PMC2910411

[35] C. B. Confavreux , R. L. Levine , G. Karsenty , Mol. Cell. Endocrinol. 2009, 310, 21. 19376193 10.1016/j.mce.2009.04.004 PMC3667507

[36] P. Ducy , R. Zhang , Cell 1997, 89, 747. 9182762 10.1016/s0092-8674(00)80257-3

[37] T. Yanai , T. Katagiri , S. Akiyama , M. Imada , T. Yamashita , H. Chiba , N. Takahashi , T. Suda , J. Bone. Miner. Metab. 2001, 19, 345. 11685649 10.1007/s007740170003

[38] D. W. Stafford , J. Thromb. Haemost. 2005, 3, 1873. 16102054 10.1111/j.1538-7836.2005.01419.x

[39] X. Lin , S. Patil , Y. G. Gao , A. Qian , Front. Pharmacol. 2020, 11, 757. 32528290 10.3389/fphar.2020.00757 PMC7264100

[40] S. Varma , J. P. Orgel , J. D. Schieber , Biophys. J. 2016, 111, 50. 27410733 10.1016/j.bpj.2016.05.038 PMC4945622

[41] L. A. Shiflett , L. M. Tiede‐Lewis , Y. Xie , Y. Lu , E. C. Ray , S. L. Dallas , Front. Cell. Dev. Biol. 2019, 7, 178. 31620436 10.3389/fcell.2019.00178 PMC6759523

[42] F. Verrecchia , A. Mauviel , Cell Signal 2004, 16, 873. 15157666 10.1016/j.cellsig.2004.02.007

[43] D. D. Smith , M. Gowen , G. R. Mundy , Endocrinology 1987, 120, 2494. 3106020 10.1210/endo-120-6-2494

[44] K. Scharffetter , M. Heckmann , A. Hatamochi , C. Mauch , B. Stein , G. Riethmuller , H. W. Ziegler‐Heitbrock , T. Krieg , Exp. Cell Res. 1989, 181, 409. 2538336 10.1016/0014-4827(89)90098-0

[45] X. Chen , J. Wang , X. Zhu , X. Yang , Y. Fan , X. Zhang , RSC Adv. 2016, 6, 102134.

[46] J. M. Wozney , V. Rosen , A. J. Celeste , L. M. Mitsock , M. J. Whitters , R. W. Kriz , R. M. Hewick , E. A. Wang , Science 1998, 242, 1528. 10.1126/science.3201241 3201241

[47] Y. Honda , T. Anada , T. Kamakura , M. Nakamura , S. Sugawara , O. Suzuki , Biochem. Biophys. Res. Commun. 2006, 345, 1155. 16716259 10.1016/j.bbrc.2006.05.013

[48] Y. Zhang , T. Böse , R. E. Unger , J. A. Jansen , C. J. Kirkpatrick , J. J. J. P. van den Beucken , Cell Tissue Res. 2017, 369, 273. 28361303 10.1007/s00441-017-2598-8 PMC5552848

[49] P. Guihard , Y. Danger , B. Brounais , E. David , R. Brion , J. Delecrin , C. D. Richards , S. Chevalier , F. Rédini , D. Heymann , H. Gascan , F. Blanchard , Stem Cells 2012, 30, 762. 22267310 10.1002/stem.1040

[50] G. E. Glass , J. K. Chan , A. Freidin , M. Feldmann , N. J. Horwood , J. Nanchahal , Proc. Natl. Acad. Sci. USA 2011, 108, 1585. 21209334 10.1073/pnas.1018501108 PMC3029750

[51] N. Ronkina , M. Gaestel , Annu. Rev. Biochem. 2022, 91, 505. 35303787 10.1146/annurev-biochem-081720-114505

[52] J. Guicheux , J. Lemonnier , C. Ghayor , A. Suzuki , G. Palmer , J. Caverzashio , J. Bone Miner. Res. 2003, 18, 2060. 14606520 10.1359/jbmr.2003.18.11.2060

[53] K. Xiao , K. Yang , J. Wang , P. Sun , H. Huang , H. Khaliq , M. A. Naeem , J. Zhong , K. Peng , Biol. Trace Elem. Res. 2019, 189, 209. 30094741 10.1007/s12011-018-1441-8

[54] K. Rutault , C. A. Hazzalin , L. C. Mahadevan , J. Biol. Chem. 2001, 276, 6666. 11076936 10.1074/jbc.M005486200

[55] H. S. Liu , C. E. Pan , Q. G. Liu , W. Yang , X. M. Liu , World J. Gastroenterol. 2003, 9, 2513. 14606087 10.3748/wjg.v9.i11.2513 PMC4656531

[56] J. Garcia , B. Lemercier , S. R. Roman , G. Rawadi , J. Biol. Chem. 1998, 273, 34391. 9852105 10.1074/jbc.273.51.34391

[57] W. S. Yang , Y. C. Park , J. H. Kim , H. R. Kim , T. Yu , S. E. Byeon , L. D. Unsworth , J. Lee , J. Y. Cho , Mediators Inflamm. 2012, 2012, 489810. 22315508 10.1155/2012/489810 PMC3270444

[58] X. Guo , X. F. Wang , Cell Res. 2008, 19, 71. 10.1038/cr.2008.302 PMC3606489 19002158

[59] Y. Q. Xiao , K. Malcolm , G. S. Worthen , S. Gardai , W. P. Schiemann , V. A. Fadok , D. L. Bratton , P. M. Henson , J. Biol. Chem. 2002, 277, 14884. 11842088 10.1074/jbc.M111718200

