# Supplementary material for: Borate‐Ion‐Stimulated Macrophages Promote Osteogenic Differentiation of Mesenchymal Stem Cells
Source: Adv Healthc Mater. 2025 Sep 9;15(2):e02570. doi: 10.1002/adhm.202502570 (PMC12805609; doi:10.1002/adhm.202502570)
Supplement: Supplementary file 1 — Supporting Information [file ADHM-15-0-s001.docx]

Supporting Information

**Borate-Ion-Stimulated Macrophages Promote Osteogenic Differentiation of Mesenchymal Stem Cells**

Kazumasa Ikedo, Hiroki Hatakeyama, Sayaka Oguri, Akiko Obata*, Toshihiro Kasuga


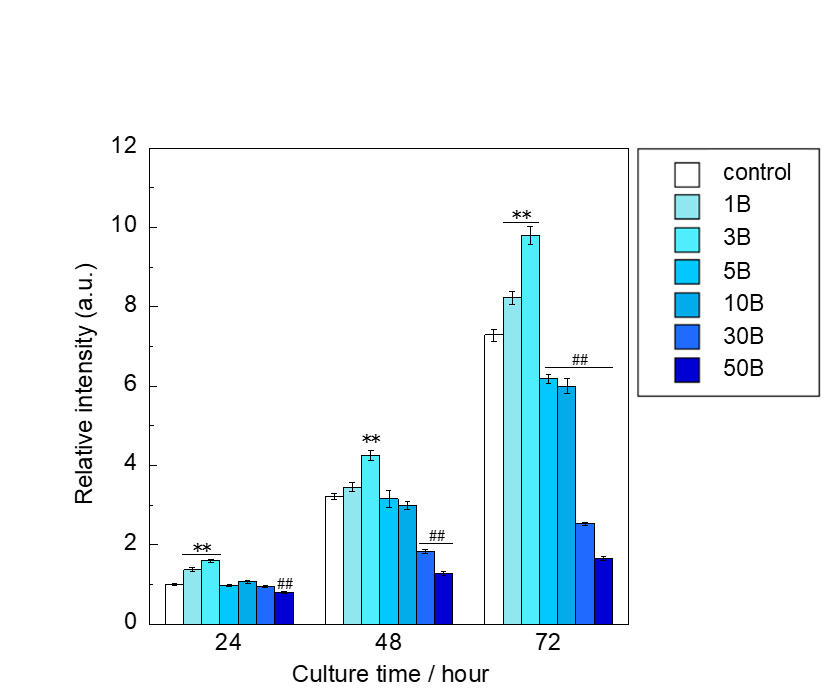


**Figure S1.** Relative metabolic activity of RAW264 cultured with B-containing medium-high. (*n* = 4, **, ##: p < 0.01 vs. control)

Figure S1. shows metabolic activity values of RAW264 cultured with B-containing medium-high. Before measuring DNA amounts, metabolic activity of the cells was measured using alamarBlue™ Cell Viability Reagent (alamarBlue, Life Technologies). First, culture medium was aspirated from each well of 96-well plate. Then, 110 µL of alamarBlue reagent, diluted 11 times with normal-medium-high, was added to each well. The samples were incubated for 4 hours to allow the reaction to proceed. After incubation, 80 µL of the reacted sample was transferred from each well to a black 96-well plate for fluorescence measurement. Fluorescence intensity was measured by a multi-mode plate reader (excitation: 500 nm, emission: 600 nm). The results for each sample were obtained as relative values, with the value of the control sample set to 1. The samples were washed with PBS after the fluorescence measurement, and the DNA amount of the samples was quantified using the method described in Section 5-3.


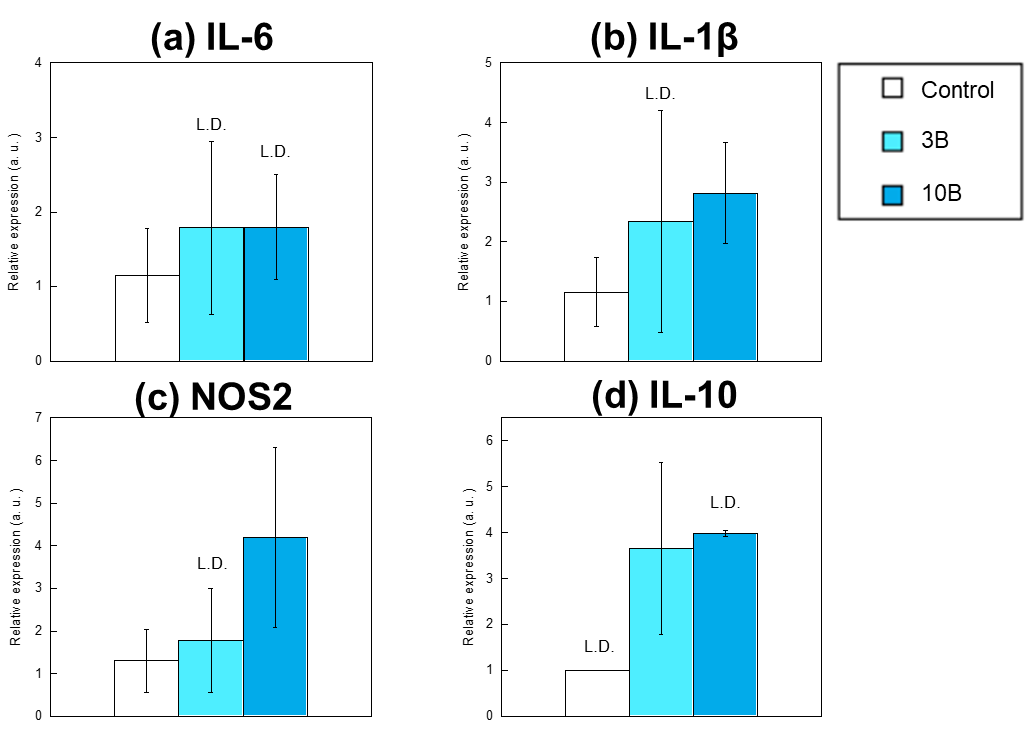


**Figure S2.** Genes related to interleukin-6 (IL-6), interleukin-1β (IL-1β), nitric oxide synthase 2 (NOS2), and interleukin-10 (IL-10) were measured by using RT-PCR. Gene expression of (a) IL-6, (b) IL-1β, (c) NOS2, and (d) IL-10 of RAW264 cultured with the B-containing mixture. (*n* = 3, L.D., values obtained from fewer than three samples).
